# Detailed Clinical Features of Deafness Caused by a Claudin-14 Variant

**DOI:** 10.3390/ijms20184579

**Published:** 2019-09-16

**Authors:** Tomohiro Kitano, Shin-ichiro Kitajiri, Shin-ya Nishio, Shin-ichi Usami

**Affiliations:** 1 Department of Otorhinolaryngology, Shinshu University School of Medicine, 3-1-1 Asahi, Matsumoto, Nagano 390-8621, Japan; tomokitano@shinshu-u.ac.jp (T.K.);; 2 Department of Hearing Implant Sciences, Shinshu University School of Medicine, 3-1-1 Asahi, Matsumoto, Nagano 390-8621, Japan

**Keywords:** tight junction, Claudin-14, *CLDN14*, hearing loss, vestibular function, cochlear implantation

## Abstract

Tight junctions are cellular junctions that play a major role in the epithelial barrier function. In the inner ear, claudins, occludin, tricellulin, and angulins form the bicellular or tricellular binding of membrane proteins. In these, one type of claudin gene, *CLDN14*, was reported to be responsible for human hereditary hearing loss, DFNB29. Until now, nine pathogenic variants have been reported, and most phenotypic features remain unclear. In the present study, genetic screening for 68 previously reported deafness causative genes was carried out to identify *CLDN14* variants in a large series of Japanese hearing loss patients, and to clarify the prevalence and clinical characteristics of DFNB29 in the Japanese population. One patient had a homozygous novel variant (c.241C>T: p.Arg81Cys) (0.04%: 1/2549). The patient showed progressive bilateral hearing loss, with post-lingual onset. Pure-tone audiograms indicated a high-frequency hearing loss type, and the deterioration gradually spread to other frequencies. The patient showed normal vestibular function. Cochlear implantation improved the patient’s sound field threshold levels, but not speech discrimination scores. This report indicated that claudin-14 is essential for maintaining the inner ear environment and suggested the possible phenotypic expansion of DFNB29. This is the first report of a patient with a tight junction variant receiving a cochlear implantation.

## 1. Introduction

### 1.1. Hearing Loss

Hearing loss (HL) is the most common sensory impairment and is diagnosed in approximately two in every 1000 children [1]. At least 60% of all childhood nonsyndromic sensorineural hearing loss is caused by genetic factors [2]. The inheritance patterns of this form of HL include autosomal recessive, autosomal dominant, X-linked, and mitochondrial. Autosomal recessive nonsyndromic hereditary HL (ARNSHL) is typically prelingual, and accounts for approximately 70% of nonsyndromic hereditary HL patients [3]. Thus far, 75 causative genes for ARNSHL have been identified [4]. One form of ARNSHL is DFNB29 (OMIM #: 614035), which is caused by variants in the *CLDN14* gene.

### 1.2. Tight Junctions in the Inner Ear

For our sound receiving process, it is necessary to convert sound vibration to nerve action potential (mechano-transduction) [5]. The center for this mechano-transduction is the cochlea, located in the inner ear. The cochlea is filled with two types of lymph fluid, the endolymph and perilymph. These fluids are completely different in their chemical composition: the perilymph resembles extracellular fluids in general [6,7] but the endolymph has the characteristics of an intracellular fluid in that it has high K^+^ and low Na^+^ concentrations [8]. Furthermore, the electrical potential of the endolymph, i.e., endocochlear potential (EP) is positive by approximately 80 to 90 mV relative to the perilymph [9,10]. It is now widely accepted that these characteristics of the endolymph (high K^+^ concentration and EP) are indispensable for cochlear hair cells to transduce acoustic stimuli into electrical signals [11]. The barrier function of the epithelial cell sheet prevents paracellular permeability, and the separation of these two fluids is essential for the maintenance of their differences in composition [12]. Tight junctions (TJs) are cellular junctions that play a major role in epithelial barrier function. In the inner ear, claudins, occludin, tricellulin, and the angulin family proteins (angulin-1/*LSR*, angulin-2/*ILDR1*, and angulin-3/*ILDR2*) form the bicellular or tricellular binding of membrane proteins. Among these, claudin-14, tricellulin, and angulin-2 are reported to be responsible for human deafness DFNB29, DFNB49, and DFNB42, respectively [13,14,15].

The claudins are a family of proteins that play a major role in epithelial barrier function, especially in bicellular junctions. There are 24 claudins that have been identified in humans thus far [13,16,17,18], with at least 10 of these reported to be expressed in the inner ear [19]. Their expression can be categorized into three types: (1) claudin-1, claudin-2, claudin-3, claudin-9, claudin-10, claudin-12, claudin-14, and claudin-18 that are expressed in multiple sites such as the organ of Corti, Reissner’s membrane, the spiral limbus, and the marginal cells of the stria vascularis; (2) claudin-8 that is expressed in all the aforementioned sites except for the organ of Corti; and (3) claudin-11 that is expressed only in the basal cells of the stria vascularis [19]. The barrier functions are speculated to be dependent on the combinations of these claudins, and previous reports have shown claudin-9, claudin-11, and claudin-14 to be critical for hearing functions [20,21,22,23,24,25], with mutations in these proteins causing deafness in humans and mice. Murine studies have indicated that claudin-11 (cldn-11) knockout mice demonstrate hearing loss due to reduced EP [20,21]. Other studies have indicated that, as a result of the rapid degeneration of cochlear hair cells shortly after birth, *Cldn9* mutant mice and *Cldn14* mutant mice demonstrate hearing loss. However, these two animal models do not display reduced EP [22,23]. The variations within these phenotypes are thought to be correlated with the multiple functions of the TJs in the inner ear. Thus, the TJs in the epithelial barriers are significantly involved in inner ear function.

### 1.3. CLDN14 Variants in Deafness

To date, nine different variants in *CLDN14* [13,26,27,28,29] have been reported to cause HL in the Pakistani, Greek, and Canadian populations. Seven of the nine variants were reported from Pakistani consanguineous families. The *CLDN14* variants are a relatively common cause of recessive hearing loss, which is responsible for 2.25% of HL patients in a Pakistani study cohort [28], whereas no pathogenic variants were reported from east Asian populations [30,31]. Although previously reported papers have shown some clinical characteristics of patients with *CLDN14* variants, the audiovestibular findings, such as progression and details of vestibular function, remain unclear. In addition, there are no reports on patients with cochlear implantation (CI), thus the outcome of CI is also unknown. In the present study, we used massively parallel DNA sequencing (MPS) to detect pathogenic variants in *CLDN14* among a large series of Japanese HL patients. 

Here, we present a patient with a novel variant in the *CLDN14* gene identified from a group of non-consanguineous HL patients and discuss additional clinical features including CI outcomes. This is the first report of a diagnosis of hearing caused by *CLDN14* in an east Asian population and in such a patient receiving a cochlear implant as intervention.

## 2. Results

### 2.1. Detected Variant

**Figure 1:**
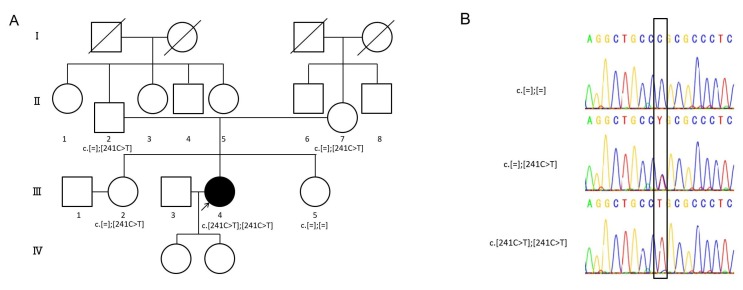
Pedigree and *CLDN14* variants of the family. (**A**) Pedigree shows autosomal recessive inherited hearing loss (HL), (**B**) the electropherograms of this family. Target genome enrichment for 68 previously reported deafness causative genes and massively parallel DNA sequencing are carried out for this proband (III-4). Sanger sequencing is used for family segregation analysis. Genetic analysis results are shown under the proband and family members.

**Figure 2:**
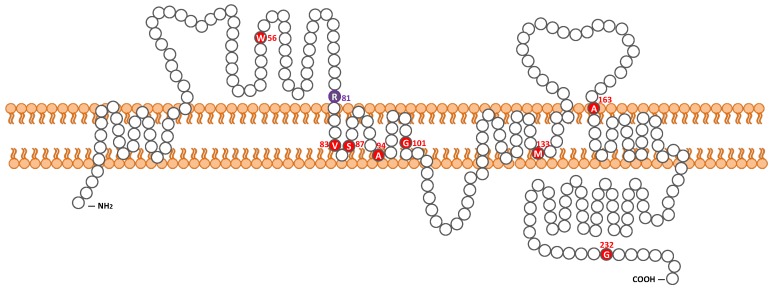
Location of pathogenic variants in Claudin-14. Red colored amino acid residues indicate previously reported claudin-14 variants. The blue colored residue indicates the positions of *CLDN14* p.Arg81His and p.Arg81Cys.

One possible disease-causing variant, *CLDN14*: NM_144492: c.241C>T, leading to p.Arg81Cys, was homozygously detected in one of 1577 probands with autosomal recessive HL (Table 1, Figure 1), whereas no pathogenic variants were found in the other 972 probands with autosomal dominant or inheritance pattern of unknown HL. At the same amino acid residue, which is within the first extracellular loop, a different missense change c.242G>A, leading to p.Arg81His, has been reported (Figure 2) [27]. No candidate variants in the other 67 deafness genes were identified in the proband. In detail, the variants in two other genes, *PTPRQ* and *EYA4*, were also detected from the proband but were not segregated. As shown in Figure 1, family segregation was confirmed by using Sanger sequencing. The variant was not identified in ExAC, gnomAD, 3.5KJPN or the 1208 Japanese exome variants, in addition to the 333 in-house Japanese normal hearing control databases.

We employed in silico software (SIFT, polyphen-2, LRT, Mutation Taster, etc.), and almost all scores indicated “damaging” (Table 1). The corresponding amino acid was well conserved across species (170/170 (100%) in vertebrates). Taken together, according to the American College of Medical Genetics (ACMG) guideline, the variant was classified as “likely pathogenic” (PS4, PM2, and PM5).

### 2.2. Clinical Findings

**Figure 3:**
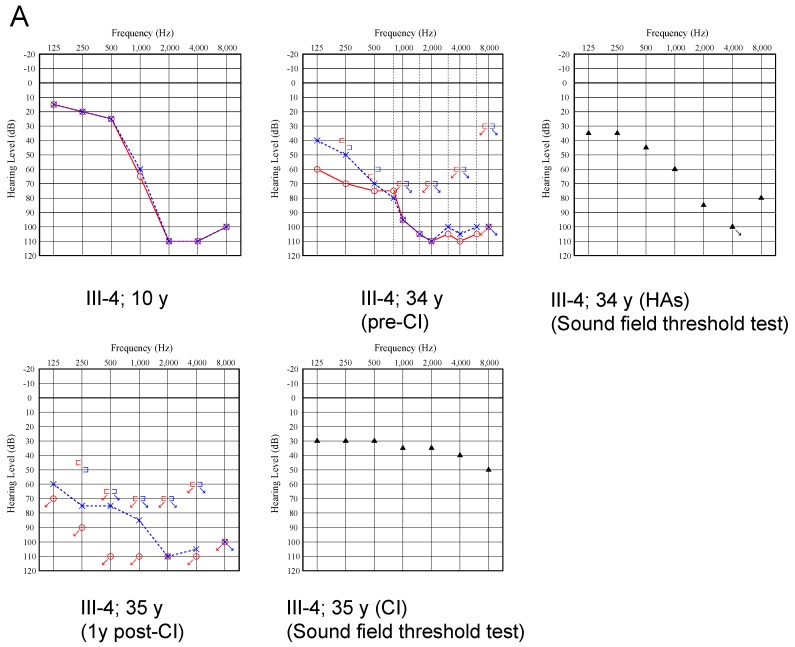

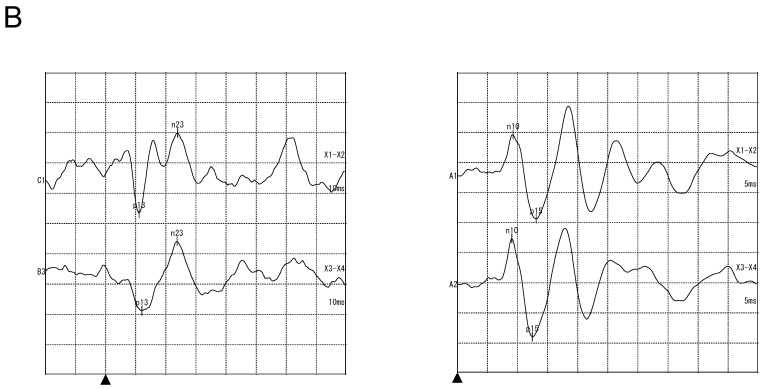
(**A**) Pure-tone audiometry (PTA) shows bilateral progressive sensorineural hearing loss in the proband. After a cochlear implantation (CI) in the right ear, sound field hearing threshold tests with a CI show 40 dBSPL. Red: right ear, Blue: left ear, Circle and Cross: Right and left ear air conduction hearing level respectively, Square bracket: Bone conduction hearing level, Triangle: both ears with intervention, Arrow: over measurement limit (**B**) There are no obvious differences between each ear in the cervical vestibular evoked myogenic potentials (right), and ocular vestibular evoked myogenic potentials (left).

The proband was a 37-year-old female. The newborn hearing screening program had not yet started at birth and there were no particular complications in the perinatal period. The female proband also passed an elementary school health checkup at six years of age, but was suspected of hearing loss at age nine and was referred to an otolaryngology clinic at a local general hospital. Pure-tone audiograms showed steep high-frequency sensorineural HL (SNHL) with an average of 77 dBHL in both ears (Figure 3A). It appeared to progress slowly, and the proband began to wear hearing aids bilaterally at 11 years of age. Over a period of 20 years, their residual hearing in the lower frequencies gradually deteriorated and hearing aids became ineffective. At the age of 34, the proband consulted our hospital for further examination. Otoscopic examination revealed a normal tympanic membrane. Computed tomography (CT) and magnetic resonance imaging (MRI) of the temporal bones showed no malformations, hearing level was approximately 100 dBHL in both ears, but the left ear showed moderate residual hearing in the lower frequencies. We performed vestibular assessment (caloric test and cervical-ocular vestibular evoked myogenic potentials, i.e., c/oVEMPs) as preoperative examinations prior to CI. The caloric test, cVEMPs, and oVEMPs represent the function of the semicircular canal, the saccule and inferior vestibular nerve, and the utricle and superior vestibular nerve, respectively. As shown in Figure 3B, all vestibular testing showed normal vestibular functions.

The proband underwent a CI (MED-EL FLEX28) in the right ear at the age 35. Sound field threshold levels were improved from 77.5 dBSPL (with hearing aids) to 37.5 dBSPL (with the cochlear implant) (Figure 3A). To evaluate speech perception outcomes, the proband underwent speech discrimination testing (using the 67S Japanese monosyllable test, presenting in 65 dBSPL). Contrary to the good improvement in sound field threshold levels, the proband’s speech perception outcomes improved from 12% to only 32% two years after receiving a CI.

## 3. Discussion

### 3.1. Frequency of CLDN14-Associated HL in the Japanese Population

We discovered a novel causative variant in the *CLDN14* gene as a cause of progressive SNHL in a Japanese patient. To date, a total of nine pathogenic variants in *CLDN14* have been reported (Table 2, Figure 2). Among them, seven were reported from Pakistan, and one each from Greece and Canada. Otherwise, according to the previous reports, no pathogenic variants of *CLDN14* have been detected in the Korean and Chinese populations [30,31]. Hence, this is the first report of a causative *CLDN14* variant in an east Asian population. 

The incidence of *CLDN14* variants was 0.04% (1/2549) among the Japanese HL patients, and 0.06% (1/1577) among the families with autosomal recessive HL in the Japanese population. In clear contrast to this, variants of *CLDN14* account for 2.25% of autosomal recessive HL in the Pakistani population. In this study, the patient was identified from a non-consanguineous family and the parents of the proband were from a geographically remote area, whereas almost all of the previous reports were from consanguineous families [13,27,28,29]. As no pathogenic *CLDN14* variants were found in our cohort, but one HL patient with a homozygous *CLDN14* variant was identified, it is necessary to consider such rare HL cases.

### 3.2. Clinical Characteristics

#### 3.2.1. Progression and Onset of DFNB29

Until Pater et al. reported the progression of HL [29], the phenotype of DFNB29 was associated with non-progressive, congenital or prelingual HL with a variable degree of severity [13,26,27,28]. In this report, serial audiograms showed hearing deterioration especially at low frequencies (125 to 500 Hz). The average threshold progression rate of HL in the low frequencies was about 1.6 dB/year. Moreover, in this case, HL in the high frequencies was already impaired from the first decade, but there may have been no problem at birth as there was no problem in articulation. According to Pater’s report, the phenotype of DFNB29 showed no hearing impairment until three years of age, and its onset occurred after age four especially in the higher frequencies. After that it progressed to almost deaf at above 2 kHz by nine years of age, therefore, it might be that our patient had a similar course. Indeed, in our case, no hearing impairment was detected at the elementary school health checkup at six years of age and it was only suspected at nine years of age. Lee et al. reported HL patients with another amino acid change, p.Arg81His [27], at the same amino acid residue. In that report, the phenotype was described as non-progressive prelingual HL. The audiogram indicated bilateral severe to profound HL with a gentle slope at 20 years of age. From our study and the previous reports, the hearing deterioration in patients with *CLDN14* remains unclear, and further study including serial audiograms is required to clarify this. 

#### 3.2.2. Vestibular Examination

We could not identify any dysfunction on the vestibular examinations. According to past reports, claudin-14 is also expressed in the vestibule of mice (such as in the sensory epithelia) [19]. Vestibular function in *Cldn14* knockout model mice has not been reported and the details remain unknown. This is the first report to mention in detail the vestibular function, including caloric testing, cVEMPs, and oVEMPs. Endolymph in cochlear is characterized by extremely high resting potential called EP, in addition to high K^+^ concentration. In contrast, in the vestibular endolymph, the K^+^ concentration is high, but no EP can be detected. Claudin-14 may be necessary for maintaining a barrier to resist EP, but not K^+^.

#### 3.2.3. Outcome of Cochlear Implantation

This is the first report showing the outcome for a CI for a *CLDN14*-associated HL patient. A CI represents the most successful neural prosthesis in clinical cases [32]. For patients with severe to profound SNHL, CI has been established as the standard therapy [33]. The implant is surgically implanted and works by transducing acoustic energy into an electrical signal, with an electrode array in the cochlea used to stimulate the surviving spiral ganglion cells of the auditory nerve [34].

As mentioned above, the patient showed relatively poor improvement in speech discrimination scores, whereas good improvement in sound field threshold levels was observed. Since the cochlear implant directly stimulates the cochlear nerve, the influence of the inner ear should be excluded. Thus, it is likely that the unsatisfactory speech discrimination was due to deficiencies in the spiral ganglion or central auditory pathway. In our previous study, *CLDN14* was expressed at the same level in the organ of Corti and spiral ganglions [35]. Including this report, there have been no cases with intellectual disability indicating central involvement; therefore, it is possible that the spiral ganglion may be involved in claudin-14 associated HL.

## 4. Materials and Methods 

### 4.1. Subjects

All procedures were approved by the Shinshu University Ethical Committee (No. 387—4 September 2012 and No. 576—2 May 2017) as well as the respective Ethical Committees of the other participating institutions described elsewhere [36] and were carried out after obtaining written informed consent from all subjects (or from their next of kin, caretaker, or guardian in the case of minors or children). A total of 2549 probands from unrelated Japanese HL families were enrolled from the 67 otolaryngology departments across Japan participating in the present study from May 2012 to September 2016. The age of the probands ranged from 0 to 79 years (mean ± SD: 22.1 ± 19.7). The hereditary patterns of the HL in the families of the probands were autosomal dominant in 602, autosomal recessive in 1577, and unknown inheritance mode in 370.

### 4.2. Variant Analysis

For the genetic analysis for this proband, we performed target genome enrichment for 68 previously reported genetic causes for deafness and MPS analysis described elsewhere [37]. In brief, amplicon libraries were prepared using an Ion AmpliSeq™ Custom Panel (Applied Biosystems, Life Technologies, Carlsbad, CA, USA), in accordance with the manufacturer’s instructions, for 68 genes reported to cause nonsyndromic hereditary HL [37]. Emulsion PCR and sequencing was performed according to the manufacturer’s instructions. The detailed protocol has been described elsewhere [38,39]. MPS was performed with an Ion Proton™ system using the Ion PI™ Hi-Q™ Sequencing 200 Kit and Ion PI™ Chip (ThermoFisher Scientific, Waltham, MA, USA) according to the manufacturers’ instructions. The sequence data were mapped against the human genome sequence (build GRCh37/hg19) with a Torrent Mapping Alignment Program. The mean depth of coverage of 68 target genes was 363.0. The percentage of each region with more than 20 times coverage (indicating the percentage of each region sequenced 20 times or more by MPS) was 95.31%. After sequence mapping, the DNA variant regions were piled up with Torrent Variant Caller plug-in software. After variant detection, their effects were analyzed using ANNOVAR software [40,41]. 

The missense, nonsense, insertion/deletion, and splicing variants were selected from among the identified variants. Variants were further selected as less than 1% of (1) the ExAC [42,43], (2) gnomAD [44], (3) 3.5KJPN [45], (4) the Human Genetic Variation Database (dataset for 1208 Japanese exome variants) [46], and (5) the 333 in-house Japanese normal hearing controls. Direct sequencing was utilized to confirm the selected variants. The pathogenicity of a variant was evaluated by ACMG (American College of Medical Genetics, Bethesda, MD, USA) standards and guidelines [47]. For missense variants, in particular, functional prediction software, including Sorting Intolerant from Tolerant (SIFT) [48], Polymorphism Phenotyping (PolyPhen2) [49], Likelihood Ratio Test (LRT) [50], Mutation Taster [51], Mutation Assessor [52], Rare Exome Variant Ensemble Learner (REVEL) [53], and Combined Annotation Dependent Depletion (CADD) [54] were used on the ANNOVAR software. Conservation of the variant site was also evaluated in 170 vertebrates from the HGMD professional [55]. Segregation analysis was performed for the proband and family members. 

### 4.3. Clinical Evaluations

The age of onset of HL, the incidence of progressive HL, and episodes of vertigo and dizziness were analyzed based on the medical charts of the probands with the *CLDN14* variant.

Pure-tone audiometry was performed to evaluate HL. The pure-tone average (PTA) was calculated from the audiometric thresholds at four frequencies (0.5, 1, 2, and 4 kHz). If an individual did not respond to the maximum hearing level at a frequency, 5 dB was added to the maximum hearing level. The severity of HL was classified into mild (PTA: 20–40 dBHL), moderate (41–70 dBHL), severe (71–95 dBHL), and profound (>95 dBHL). The audiometric configurations were categorized as low-frequency, mid-frequency, high-frequency (gently or steeply sloping), flat, and deaf [56]. 

The vestibular examination findings, including caloric testing and the measurement of cervical and ocular vestibular evoked myogenic potentials (c/oVEMPs), were analyzed. Caloric testing involved the measurement of the maximum slow phase velocity (SPV) by cold water irrigation (20 °C, 5 mL, 20 s). We defined a maximum SPV value below 10 deg/s as representing areflexia and a value between 10 and 20 deg/s as representing hyporeflexia. For cVEMPs testing, electromyography (EMG) was performed using a pair of surface electrodes mounted on the upper half and sternal head of the sternocleidomastoid muscle, respectively. The electrographic signal was recorded using a Neuropack evoked potential recorder (Nihon Kohden Co Ltd., Tokyo, Japan). Clicks lasting for 0.1 ms at 105 dBnHL were presented through a headphone. The stimulation rate was 5 Hz, the bandpass filter intensity was 20 to 2000 Hz, and the analysis time was 50 ms. The responses to 100 stimuli were averaged twice. The oVEMPs testing was measured by bone-conductive vibration (BCV). The BCV was delivered in 4 ms tone bursts of 500 Hz vibration (rise and fall time = 1 ms and plateau time = 2 ms) by using a hand-held 4810 mini-shaker (Bruel and Kjaer, Naerum, Denmark), which was placed on the midline (Fz) of the forehead. The active electrode was located over the inferior orbital margin and a reference electrode was placed 2 cm below the active electrode. The ground electrode was placed on the chin. The patients laid in a supine position on a bed and looked up at an angle of approximately 30 degrees above straight ahead during the recording. The signals were amplified and bandpass filtered between 20 and 2000 Hz. The stimulus intensity was 115 dB force level, 500 Hz with an analysis time of 40 ms, and 50 responses were averaged for each run. The VEMPs asymmetry was calculated as follows: asymmetry ratio (AR) = (larger amplitude – smaller amplitude) × 100/(larger amplitude + smaller amplitude). In this study, an asymmetry ratio of >30% was defined as a decreased reaction and no reaction in amplitude VEMPs as absent.

Intervention for HL, including the use of hearing aids or cochlear implants, was investigated. To evaluate speech perception outcomes, speech discrimination scores (using the 67S Japanese monosyllable test) were used. The subjects sat 1 m away from the sound source facing zero-degree azimuth, and recorded monosyllable words in quiet were presented in the sound field at 65 dBSPL.

## 5. Conclusions

We present a patient with a novel variant in the *CLDN14* gene identified from a non-consanguineous family. This is the first report of *CLDN14*-associated HL in an east Asian population. Serial audiograms indicated high-frequency hearing loss type, and the deterioration gradually spread to other frequencies, finally resulting in deafness. The patient showed normal vestibular function for caloric testing, cVEMPs, and oVEMPs. This is also the first report of an HL patient with a tight junction variant receiving a CI. The CI improved the proband’s sound field threshold levels, but not their speech discrimination scores. This information contributes to our understanding, diagnosis, and treatment of HL caused by TJ disorders.

## Figures and Tables

**Table 1:** Possible causative variant identified in this study.

		Prediction Score								Allele Frequency in Controls		
Nucleotide Change	Amino Acid Change	SIFT *	PolyPhen2_HVAR *	LRT *	Mut_Taster *	Mut_Assessor *	REVEL *	CADD	Evolutional Conservation **	ExAC	gnomAD	3.5kJPN
c.241C > T	p.R81C	0.78	0.88	0.84	0.81	0.98	0.89	27.6	Yes (100%)	0.000042	0.000037	0

* The prediction scores of each algorithm included on the ANNOVAR software were converted from the original scoring system. Scores closer to 1.0 indicated the mutation was more damaging, and those closer to 0 indicated they were more tolerant. ** Conserved in 170 vertebrates from the HGMD professional.

**Table 2:** Summary of clinical features associated with *CLDN14* variants.

			HL		Pure-Tone Audiometry						
Nucleotide Change	Genotype	Amino Acid Change	Onset	Progression	Severity of HL	Audiometric Configuration	Consanguineous Marriage	Vestibuler Function	Hearing Intervention	Family Origin	Reference
c.167G > A	homozygote	p.Trp56 *	NA	No	severe to profound	gently sloping	Yes	NA	NA	Pakistan	Lee et al. 2011 [27]
c.241C > T	homozygote	p.Arg81Cys	9 y.o.	Yes	Severe	steeply sloping	No	Normal	CI	Japan	This study
c.242G > A	homozygote	p.Arg81His	NA	No	severe to profound	gently sloping	Yes	NA	NA	Pakistan	Lee et al. 2011 [27]
c.254T > A	homozygote	p.Val85Asp	prelingual	No	moderate to profound	HF	Yes	NA	NA	Pakistan	Wilcox et al. 2001 [13], Bashir et al. 2013 [28]
c.259_260TC > AT	homozygote	p.Ser87Ile	prelingual	No	severe to profound	flat	Yes	NA	NA	Pakistan	Bashir et al. 2013 [28]
c.281C > T	homozygote	p.Ala94Val	NA	No	moderate to profound	flat, DE	Yes	NA	NA	Pakistan	Bashir et al. 2013 [28]
c.301G > A	homozygote	p.Gly101Arg	NA	NA	NA	NA	NA	NA	NA	Greece	Wattenhofer et al. 2005 [26]
c.398delT	homozygote	p.Met133Argfs *24	congenital	NA	moderate to profound	gently sloping	Yes	NA	NA	Pakistan	Wilcox et al. 2001 [13], Bashir et al. 2013 [28]
c.488C > T	homozygote	p.Ala163Val	5–7 y.o.	Yes	mild to severe	steeply sloping	Yes	Normal	HA	Canada	Pater et al. 2016 [29]
c.694G > A	homozygote	p.Gly232Arg	NA	No	severe to profound	gently sloping	Yes	NA	NA	Pakistan	Lee et al. 2011 [27]

Abbreviations: HF, high-frequency hearing loss; DE, deaf; CI, cochlear implant; HA, hearing aid; N/A, not available.

## Data Availability

The sequencing data are available in the DDBJ databank of Japan (Accession number: JGAS00000000191).

## Abbreviations

HL	Hearing loss
ARNSHL	Autosomal recessive nonsyndromic hereditary hearing loss
EP	Endocochlear potential
TJ	Tight junction
CI	Cochlear implantation
MPS	Massively parallel DNA sequencing
PTA	Pure-tone average
cVEMPs	Cervical vestibular evoked myogenic potentials
oVEMPs	Ocular vestibular evoked myogenic potentials
SNHL	Sensorineural hearing loss
dBSPL	Decibel sound pressure level
dBHL	Decibel hearing level

## References

[1] Morton C.C., Nance W.E. (2006). Newborn Hearing Screening—A Silent Revolution. N. Engl. J. Med..

[2] Smith R.J.H., Bale J.F., White K.R. (2005). Sensorineural hearing loss in children. Lancet.

[3] Hilgert N., Smith R.J.H., Van Camp G. (2009). Forty-six genes causing nonsyndromic hearing impairment: Which ones should be analyzed in DNA diagnostics?. Mutat. Res..

[4] Hereditary Hearing Loss Homepage. https://hereditaryhearingloss.org/.

[5] Richardson G.P., de Monvel J.B., Petit C. (2011). How the Genetics of Deafness Illuminates Auditory Physiology. Annu. Rev. Physiol..

[6] Wangemann P., Schacht J., Dallos P., Popper A.N., Fay R.R. (1996). Homeostatic Mechanisms in the Cochlea. The Cochlea.

[7] Ferrary E., Sterkers O. (1998). Mechanisms of endolymph secretion. Kidney Int..

[8] Sterkers O., Ferrary E., Amiel C. (1988). Production of inner ear fluids. Phys. Rev..

[9] Tasaki I., Spyroupoulos C.S. (1959). Stria vascularis as source of endocochlear potential. J. Neurophysiol..

[10] Konishi T., Hamrick P.E., Walsh P.J. (1978). Ion transport in guinea pig cochlea. I. Potassium and sodium transport. Acta Otolaryngol..

[11] Hudspeth A.J. (1989). How the ear’s works work. Nature.

[12] Nunes F.D., Lopez L.N., Lin H.W., Davies C., Azevedo R.B., Gow A., Kachar B. (2006). Distinctsubdomain organization and molecular composition of a tight junction with adherens junction features. J. Cell Sci..

[13] Wilcox E.R., Burton Q.L., Naz S., Riazuddin S., Smith T.N., Ploplis B., Belyantseva I., Ben-Yosef T., Liburd N.A., Morell R.J. (2001). Mutations in the gene encoding tight junction claudin-14 cause autosomal recessive deafness DFNB29. Cell.

[14] Riazuddin S., Ahmed Z.M., Fanning A.S., Lagziel A., Kitajiri S., Ramzan K., Khan S.N., Chattaraj P., Friedman P.L., Anderson J.M. (2006). Tricellulin Is a Tight-Junction Protein Necessary for Hearing. Am. J. Hum. Genet..

[15] Borck G., Rehman A.U., Lee K., Pogoda H., Kakar N., von Ameln S., Grillet N., Hildebrand M.S., Ansar M., Basit S. (2011). Loss-of-Function Mutations of ILDR1 Cause Autosomal-Recessive Hearing Impairment DFNB42. Am. J. Hum. Genet..

[16] Furuse M., Fujita K., Hiiragi T., Fujimoto K., Tsukita S. (1998). Claudin-1 and -2: Novel integral membrane proteins localizing at tight junctions with no sequence similarity to occludin. J. Cell Biol..

[17] Elkouby-Naor L., Ben-Yosef T. (2010). Functions of claudin tight junction proteins and their complex interactions in various physiologicalsystems. Int. Rev. Cell Mol. Biol..

[18] Gunzel D., Fromm M. (2012). Claudins and other tight junction proteins. Compr. Physiol..

[19] Kitajiri S.-I., Furuse M., Morita K. (2004). Expression patterns of claudins, tight junction adhesion molecules, in the inner ear. Hear. Res..

[20] Gow A., Davies C., Southwood C.M., Frolenkov G., Chrustowski M., Ng L., Yamauchi D., Marcus D.C., Kachar B. (2004). Deafness in Claudin 11-null mice reveals the critical contribution of basal cell tight junctions to stria vascularis function. J. Neurosci..

[21] Kitajiri S., Miyamoto T., Mineharuetal A. (2004). Compartmentalization established by claudin-11-based tight junctions in stria vascularis is required for hearing through generation of endocochlear potential. J. Cell Sci..

[22] Ben-Yosef T., Belyantseva I.A., Saunders T.L., Hughes E.D., Kawamoto K., Van Itallie C.M., Beyer L.A., Halsey K., Gardner D.J., Wilcox E.R. (2003). Claudin 14 knockout mice, a model for autosomal recessive deafness DFNB29, are deaf due to cochlear hair cell degeneration. Hum. Mol. Genet..

[23] Nakano Y., Kim S.H., Kim H.M., Sanneman J.D., Zhang Y., Smith R.J., Marcus D.C., Wangemann P., Nessler R.A., Banfi B. (2009). A claudin-9—Based ion permeability barrier is essential for hearing. PLoS Genet..

[24] Matter K., Balda M.S. (2003). Signalling to and from tight junctions. Nat. Rev. Mol. Cell Biol..

[25] Schneeberger E.E., Lynch R.D. (2004). The tight junction: A multifunctional complex. Am. J. Physiol. Cell Physiol..

[26] Wattenhofer M., Reymond A., Falciola V., Charollais A., Caille D., Borel C., Lyle R., Estivill X., Petersen M.B., Meda P. (2005). Different mechanisms preclude mutant CLDN14 proteins from forming tight junctions in vitro. Hum. Mutat..

[27] Lee K., Ansar M., Andrade P.B., Khan B., Santos-Cortez R.L.P., Ahmad W., Leal S.M. (2012). Novel CLDN14 mutations in Pakistani families with autosomal recessive non-syndromic hearing loss. Am. J. Med. Genet. A.

[28] Bashir Z.-E.-H., Latief N., Belyantseva I.A., Iqbal F., Amer Riazuddin S., Khan S.N., Friedman T.B., Riazuddin S., Riazuddin S. (2013). Phenotypic variability of CLDN14 mutations causing DFNB29 hearing loss in the Pakistani population HHS Public Access. J. Hum. Genet..

[29] Pater J.A., Benteau T., Griffin A., Penney C., Stanton S.G., Predham S., Kielley B., Squires J., Zhou J., Li Q. (2017). A common variant in CLDN14 causes precipitous, prelingual sensorineural hearing loss in multiple families due to founder effect. Hum. Genet..

[30] Kim M.-A., Kim Y.-R., Sagong B., Cho H.-J., Bae J.W., Kim J., Lee J., Park H.-J., Choi J.Y., Lee K.-Y. (2014). Genetic Analysis of Genes Related to Tight Junction Function in the Korean Population with Non-Syndromic Hearing Loss. PLoS ONE.

[31] Lu Y., Yao J., Wei Q., Xu J., Xing G., Cao X. (2018). Genetic analysis of CLDN14 in the Chinese population affected with non-syndromic hearing loss. Int. J. Pediatr. Otorhinolaryngol..

[32] Roche J.P., City I., Hansen M.R. (2016). On the Horizon: Cochlear Implant Technology. Otolaryngol. Clin. North Am..

[33] Wilson B.S. (2014). Getting a decent (but sparse) signal to the brain for users of cochlear implants. Hear. Res..

[34] Deep N.L., Dowling E.M., Jethanamest D., Carlson M.L. (2019). Cochlear Implantation: An Overview. J. Neurol. Surg. Part B.

[35] Nishio S., Takumi Y., Usami S. (2017). Laser-capture micro dissection combined with next-generation sequencing analysis of cell type-specific deafness gene expression in the mouse cochlea. Hear. Res..

[36] Nishio S.Y., Usami S.I. (2015). Deafness Gene Variations in a 1120 Nonsyndromic Hearing Loss Cohort: Molecular Epidemiology and Deafness Mutation Spectrum of Patients in Japan. Ann. Otol. Rhinol. Laryngol..

[37] Kitano T., Miyagawa M., Nishio S.-Y., Moteki H., Oda K., Ohyama K., Miyazaki H., Hidaka H., Nakamura K.-I., Murata T. (2017). POU4F3 mutation screening in Japanese hearing loss patients: Massively parallel DNA sequencing-based analysis identified novel variants associated with autosomal dominant hearing loss. PLoS ONE.

[38] Miyagawa M., Nishio S.Y., Ikeda T., Fukushima K., Usami S.I. (2013). Massively Parallel DNA Sequencing Successfully Identifies New Causative Mutations in Deafness Genes in Patients with Cochlear Implantation and EAS. PLoS ONE.

[39] Nishio S.Y., Moteki H., Usami S.I. (2018). Simple and efficient germline copy number variant visualization method for the Ion AmpliSeq^TM^ custom panel. Mol. Genet. Genomic Med..

[40] Chang X., Wang K. (2012). wANNOVAR: Annotating genetic variants for personal genomes via the web. J. Med. Genet..

[41] Wang K., Li M., Hakonarson H. (2010). ANNOVAR: Functional annotation of genetic variants from high-throughput sequencing data. Nucleic Acids Res..

[42] Abboud H.E., Abecasis G., Aguilar-Salinas C.A., Arellano-Campos O., Atzmon G., Aukrust I., Barr C.L., Bell G.I., Bergen S., Bjørkhaug L. (2016). Analysis of protein-coding genetic variation in 60,706 humans. Nature.

[43] The Exome Aggregation Consortium Database (ExAC). http://exac.broadinstitute.org/.

[44] The Genome Aggregation Database (gnomAD). https://gnomad.broadinstitute.org/.

[45] Integrative Japanese Genome Variation Database (3.5KJPN). https://ijgvd.megabank.tohoku.ac.jp/statistics/statistics-3.5kjpn-all.

[46] Narahara M., Higasa K., Nakamura S., Tabara Y., Kawaguchi T., Ishii M., Matsubara K., Matsuda F., Yamada R. (2014). Large-scale East-Asian eQTL mapping reveals novel candidate genes for LD mapping and the genomic landscape of transcriptional effects of sequence variants. PLoS ONE.

[47] Richards S., Aziz N., Bale S., Bick D., Das S., Gastier-Foster J., Grody W.W., Hegde M., Lyon E., Spector E. (2015). Standards and guidelines for the interpretation of sequence variants: A joint consensus recommendation of the American College of Medical Genetics and Genomics and the Association for Molecular Pathology. Genet. Med..

[48] Kumar P., Henikoff S., Ng P.C. (2009). Predicting the effects of coding non-synonymous variants on protein function using the SIFT algorithm. Nat. Protoc..

[49] Adzhubei I.A., Schmidt S., Peshkin L., Ramensky V.E., Gerasimova A., Bork P., Kondrashov A.S., Sunyaev S.R. (2010). A method and server for predicting damaging missense mutations. Nat. Methods.

[50] Chun S., Fay J.C. (2009). Identification of deleterious mutations within three human genomes. Genome Res..

[51] Schwarz J.M., Rödelsperger C., Schuelke M., Seelow D. (2010). MutationTaster evaluates disease-causing potential of sequence alterations. Nat. Methods.

[52] Reva B., Antipin Y., Sander C. (2011). Predicting the functional impact of protein mutations: Application to cancer genomics. Nucleic Acids Res..

[53] Ioannidis N.M., Rothstein J.H., Pejaver V., Middha S., McDonnell S.K., Baheti S., Musolf A., Li Q., Holzinger E., Karyadi D. (2016). REVEL: An Ensemble Method for Predicting the Pathogenicity of Rare Missense Variants. Am. J. Hum. Genet..

[54] Kircher M., Witten D.M., Jain P., O’Roak B.J., Cooper G.M., Shendure J. (2014). A general framework for estimating the relative pathogenicity of human genetic variants. Nat. Genet..

[55] The Human Gene Mutation Database Professional (HGMD). http://www.hgmd.cf.ac.uk/.

[56] Mazzoli M., Van Camp G., Newton V., Giarbini N., Declau F., Parving A. (2003). Recommendations for the Description of Genetic and Audiological Data for Families with Nonsyndromic Hereditary Hearing Impairment. Audiol. Med..

